# Testing Pooled Specimens With the Abbott Alinity m STI Assay: Four Years’ Experience

**DOI:** 10.7759/cureus.86110

**Published:** 2025-06-16

**Authors:** Maria Angeles Argudín, Ahalieyah Anantharajah, Antoine Mairesse, Anaïs Scohy, Giulia Zorzi, Alexia Verroken, Hector Rodriguez-Villalobos, Benoît Kabamba Mukadi

**Affiliations:** 1 Department of Microbiology, Cliniques Universitaires Saint-Luc, Université Catholique de Louvain, Brussels, BEL; 2 Department of Infectious Diseases, Laboratoire-Biologie Clinique, Cliniques de l’Europe, Brussels, BEL

**Keywords:** chlamydia trachomatis, inhibition, mycoplasma genitalium, neisseria gonorrhoeae, pooled specimens

## Abstract

Background: The Alinity m STI assay (Abbott Molecular, Des Plaines, IL, USA) is designed for the qualitative detection of *Chlamydia trachomatis* (CT), *Neisseria gonorrhoeae* (NG), *Mycoplasma genitalium* (MG), and *Trichomonas vaginalis* (TV) in individual collected samples. Pooled specimens are not recommended due to the high risk of inhibition.

Methods: We performed a two-part study, including a preliminary validation on 13 well-known positive samples before and after pooling, as well as a retrospective analysis of 5324 pooled specimens collected between 2021 and 2024. All samples were tested for CT/NG detection, and a subset (n = 277) for MG detection on the Alinity m STI system.

Results: The preliminary validation showed consistent results without inhibition. Out of 5324 specimens of the retrospective analysis for CT/NG detection, 966 pooled samples (18.1%) were positive: 363 (6.8%) for CT, 479 (9.0%) for NG, and 124 (2.3%) for both CT and NG. Only 95 (1.8%) tests were completely inhibited. A total of 277 specimens were additionally tested for MG. Pooled specimens also allowed MG detection (n = 195; 70.4%) with few inhibited results (n = 3; 1.0%).

Conclusions: This study proves that pooled specimens are rarely inhibited and can be helpful for laboratories where the number of specimens per patient is restricted.

## Introduction

The Alinity m STI assay (references: 09N17-090, 09N17-091; Abbott Molecular, Des Plaines, IL, USA) is an *in vitro* reverse transcription real-time quantitative polymerase chain reaction (RT-qPCR) assay for use with the automated Alinity m System for the qualitative detection of *Chlamydia trachomatis* (CT), *Neisseria gonorrhoeae* (NG), *Mycoplasma genitalium* (MG), and *Trichomonas vaginalis* (TV) [1,2]. The assay also includes two additional targets for monitoring the inhibition and quality of the samples: internal control (IC) and cellular control (CC). The two versions of the kit are the same product, but are validated by the company in different specimens. Version 09N17-090 was the first to be introduced in the market [1]. After a few years, the company validated additional specimens and released an updated version (09N17-091) with a revised insert [2]. The new reference 09N17-091 officially fully replaced the old reference as of February 2024. Both versions of the assay can be performed on a large variety of specimens, including endocervical swab specimens, clinician-collected vaginal swab specimens, self-collected vaginal swab specimens, gynaecological specimens, female urine, and male urine [1,2]. The 09N17-091 version of the assay has also been approved for the testing of oropharyngeal and rectal swab specimens [2]. Nevertheless, in both versions of the assay, the use of a transport tube containing multiple swabs or a combination of swabs and urine is not recommended [1,2]. The company recommends the use of a specimen with a transport tube. This recommendation is based on the high risk of inhibition by pooling the specimens. Different samples (e.g., rectal swabs and urine samples) may contain inhibitors that affect nucleic acid amplification tests [3,4]. Testing different specimens per patient can incur high costs for laboratories and patients in some healthcare systems. In this study, we present our four-year experience in testing pooled specimens for sexually transmitted infection (STI) detection using the Alinity m STI assay. To our knowledge, this is the first study evaluating the Alinity m STI assay with pooled specimens collected from multiple anatomical sites in routine clinical practice.

## Materials and methods

Definitions, study design, and samples

Pooled specimens refer to a combination of samples collected from different anatomical sites (urine, vaginal, anorectal, or oropharyngeal) from the same individual, which are mixed together and tested as a single sample. A two-part study was conducted using pooled specimens. Firstly, a preliminary validation of pooled specimens was performed using 13 samples (from different patients) collected at the Cliniques Universitaires Saint-Luc (CUSL), Brussels, Belgium, in 2019. These samples were confirmed to be positive for CT (n = 10) or NG (n = 3) by using the Abbott Real-Time CT/NG assay (reference: 08L07-091, Abbott Molecular) in the m2000sp/rt system [5]. The individual specimens were mixed with negative specimens from the same patient in Alinity m Multi-Collect Specimen Collection Kit (Abbott Molecular) tubes [6] and tested for CT/NG using the Alinity m STI assay (09N17-090) on the Alinity m System. Subsequently, different single specimens from the same patient were pooled by the laboratory staff into a former multicollector tube and tested using the Alinity m STI assay. This approach is known as ex-post pooling, which involves combining the transport media from anorectal, oropharyngeal, and urine specimens into a single pooled sample [3].

The second part of the study involved a retrospective analysis of the pooled specimens (n = 5324) collected from 1479 patients in Alinity m Multi-Collect Specimen Collection Kit tubes between 2021 and 2024 at the CUSL. We transitioned from kit 09N17-090 to kit 09N17-091 at the end of January 2024. Urine and swab samples were collected according to the manufacturer’s instructions [6] and combined into the same tube immediately after collection by trained medical personnel. This approach is known as ex-ante pooling, which involves placing the original single-site specimens into a tube containing transport medium [3]. The samples were examined for CT and NG detection using the Alinity m STI assay on an Alinity m system [1,2]. A subset of pooled specimens (n = 277) was used for MG detection. TV detection was rarely requested and was therefore excluded from this study. All specimens were anonymized, and laboratory data were collected retrospectively. The study was performed in accordance with the principles of Good Clinical Practice and conducted in adherence with the Declaration of Helsinki. Ethical approval for this retrospective research has been granted by the review board of the local ethical committee (Comité d'Ethique Hospitalo-Facultaire) at the Cliniques Universitaires Saint-Luc and Université Catholique de Louvain (reference: 2025/09MAR/104).

Assay platforms

The m2000sp/rt system is a molecular diagnostics platform from Abbott Molecular. Alinity m (Abbott Molecular), a new, fully automated, random-access molecular diagnostic analyzer, was launched in 2019. Sample processing using the Abbott RealTime CT/NG assay (reference: 08L07-091) on the m2000sp/rt system and the Alinity m STI assay (references: 09N17-090, 09N17-091) on the Alinity m system was performed according to the manufacturer’s instructions [1,2,5]. The Abbott RealTime CT/NG assay employs polymerase chain reaction (PCR) to amplify and detect dual cryptic plasmid targets for CT, as well as a specific region of the Opa gene of NG, along with an IC. Positive results with this system are determined based on threshold cycle (Ct) values. The Alinity m STI assay utilizes RT-qPCR to amplify and detect CT ribosomal RNA sequences, NG Opa gene DNA sequences, TV ribosomal RNA sequences, and MG ribosomal RNA sequences. The assay also detects armored RNA (pumpkin) as an IC and the human ß-globin gene (DNA) as a CC. The threshold cycle is defined by the Alinity m System as the cycle number (CN) at which the PCR fluorescence signal reaches a fixed detection threshold above the baseline fluorescence level. While conceptually similar to Ct values used in other quantitative PCR (qPCR) platforms, CN values are determined using Abbott’s proprietary algorithms and adaptive thresholding methods, which may result in slight numerical differences when compared to Ct values from other systems. For each reaction, a defined and consistent amount of IC is included to monitor for the presence of PCR inhibitors. The IC is an RNA sequence unrelated to the assay’s target sequences and ensures amplification performance. All specimens must fall within a defined IC CN validity range. The CC, which detects an endogenous human DNA sequence, serves to assess sample adequacy, sample extraction, and amplification efficiency. The CC CN value must be equal to or below a defined cutoff. A flag or message code is displayed when the IC CN value or the CC CN value of a specimen or control exceeds the established range. For positive specimens, if the IC CN or CC CN is out of range, but the analyte(s) in that sample is positive, the sample will yield a positive result. An IC flag or CC flag will be reported next to the positive result. For negative specimens, if the IC CN or CC CN is out of range and the analyte(s) in that sample is not positive, no result will be reported for the analyte(s), and a message code will be generated. Therefore, the test was considered inhibited, as it was negative for all targets and either the IC or the CC failed.

Statistical methods

For the preliminary evaluation, we compared the Alinity CN values. The difference, mean, standard deviation (SD), and percent coefficient of variation (%CV) were calculated. A simple statistical analysis based on percentage values was performed for the retrospective analysis.

## Results

Preliminary validation

Individual specimens were positive for the expected targets in the Alinity m (Table 1). The cycle threshold values from the m2000 system and the CN values from the Alinity m were not directly comparable because of the dilution effect in the Alinity m Multi-Collect Specimen Collection Kit tubes. Pooled specimens were positive for the expected targets. The qualitative results remained consistent despite the variations in the CN values between the individual and pooled specimens (standard deviations ranging from 0.13 to 4.12). No tests were inhibited (i.e., there were no failures for the CI and CC controls) in the Alinity m system.

**Table 1:** Validation of CT/NG detection with the Alinity m STI assay on 13 pooled specimens. CN: cycle number; Ct: cycle threshold; CT: *Chlamydia trachomatis*; CV: variation coefficient; ID: identification; NG: *Neisseria gonorrhoeae*; SD: standard deviation. ^a^ The variation coefficient (CV) percentage was calculated using the formula: SD/average * 100.

Sample ID	Test on individual specimens				Test on mixed specimens			CN comparison			
	Original specimen	Positive target	Ct m2000	CN Alinity m	Specimen(s) pooled in the original specimen	Positive target	CN Alinity m	Difference	Average	SD	CV% ^a^
28731304	Urine	CT	6.22	30.68	Rectal swab	CT	35.59	-4.91	33.14	3.47	10.48
28711078	Urine	CT	12.05	30.60	Oropharyngeal swab	CT	30.25	0.35	30.43	0.25	0.81
28697432	Urine	CT	2.25	30.80	Oropharyngeal swab. Rectal swab	CT	35.85	-5.05	33.33	3.57	10.72
28719466	Urine	CT	6.95	31.37	Rectal swab	CT	33.01	-1.64	32.19	1.16	3.60
28702905	Urine	CT	9.81	31.14	Rectal swab	CT	31.42	-0.28	31.28	0.20	0.63
28747506	Urine	CT	1.57	31.24	Rectal swab	CT	36.20	-4.96	33.72	3.51	10.40
28753798	Urine	CT	7.61	29.04	Rectal swab	CT	32.06	-3.02	30.55	2.14	6.99
28792751	Urine	CT	6,73	30.76	Oropharyngeal swab. Rectal swab	CT	36.59	-5.83	33.68	4.12	12.24
28736005	Rectal swab	CT	10.47	31.47	Urine	CT	26.15	5.32	28.81	3.76	13.06
28713869	Rectal swab	CT	1.98	33.49	Urine	CT	32.67	0.82	33.08	0.58	1.75
28755527	Urine	NG	17.66	20.32	Rectal swab	NG	21,99	-1,67	21,16	1,18	5,58
28754636	Urine	NG	5.15	31.40	Rectal swab	NG	28.92	2.48	30.16	1.75	5.81
28713868	Oropharyngeal swab	NG	0.43	35.18	Rectal swab	NG	35.00	0.18	35.09	0.13	0.36

Retrospective analysis of pooled specimens

Most pooled specimens were urine combined with oropharyngeal and rectal swabs (n = 4966; 93.3%), followed by urine combined with oropharyngeal swabs (n = 167; 3.1%). A few other pooled specimens were tested (Table 2). Of the 5324 total specimens, 966 pooled samples (18.1%) were positive: 363 (6.8%) for CT, 479 (9.0%) for NG, and 124 (2.3%) for both CT and NG. Only 95 (1.8%) tests were completely inhibited. Fourteen additional tests (0.7%) showed partial inhibition: 12 were inhibited for CT, though 11 of these were positive for NG; two were inhibited for NG but tested positive for CT. Additionally, two tests inhibited for CT/NG yielded positive MG results. Pooled specimens also allowed for MG detection (n = 195; 70.4%), with a few inhibited results (n = 3; 1.0%) in the 254 samples tested for MG.

**Table 2:** Overall results with the Alinity m STI assay of 5324 pooled specimens recovered between 2021 and 2024. CN: cycle number; CT:* Chlamydia trachomatis*; NA: not applicable; NG: N*eisseria gonorrhoeae*; MG: *Mycoplasma genitalium*; OR: oropharyngeal swab + rectal swab; OV: oropharyngeal swab + vaginal swab; ORV: oropharyngeal swab + rectal swab + vaginal swab; UO: urine + oropharyngeal swab; UOR: urine + oropharyngeal swab + rectal swab; UORV: urine + oropharyngeal swab + rectal swab + vaginal swab; UOV: urine + oropharyngeal swab + vaginal swab; UR: urine + rectal swab; URV: urine + rectal swab + vaginal swab; UV: urine + vaginal swab. ^a^ The test failed for internal control (IC) and/or cellular control (CC). ^b^ A positive NG sample gave an inhibited result for CT/MG. ^c^ Two CT-positive samples gave an inhibited result for NG. ^d^ Nine NG-positive samples gave an inhibited result for CT. ^e^ Four samples were CT/NG/MG positive, 20 were CT/MG positive, 14 were NG/MG positive, 155 were MG positive only, and two MG positive gave an inhibited result for CT/NG. ^f ^Two samples were inhibited for CT, NG, and MG detection.

Pooled specimen	CT/NG testing							Additional testing		
	No. of samples of CT/NG tested	No. of positive (range CN)			No. of inhibited tests ^a^			No. of samples of MG tested	No. of positive MG (range CN)	No. of inhibited tests for MG ^a^
		CT	NG	CT+NG	Only for CT	Only for NG	For both CT/NG			
OR	30	2 (30.17-30.79)	4 (19.54-39.61)^b^	0	2^b^	0	1	2	0	1^b^
ORV	8	0	0	0	0	0	0	0	N/A	N/A
OV	5	0	0	0	0	0	0	0	N/A	N/A
UO	167	3 (24.28-35.24)	13 (21.74-39.77)	0	0	0	2	3	0	0
UOR	4966	352 (16.25-38,79)^c^	459 (16.90-40.32)^d^	124 (CT: 21.04-38.32; NG: 15.14-38.14)	10^d^	2^c^	89	264	195 (8.95-40.34) ^e^	2^f^
UORV	15	1 (30.43)	0	0	0	0	0	0	N/A	N/A
UOV	11	0	1 (31.06)	0	0	0	0	1	0	0
UR	23	2 (27.76-32.32)	1 (29.08)	0	0	0	3	0	N/A	N/A
URV	7	1 (20.23)	1 (26.34)	0	0	0	0	0	N/A	N/A
UV	92	2 (22.50-24.70)	0	0	0	0	0	7	0	0

## Discussion

Literature on the Alinity m STI assay is scarce [7-12]. All published research has been conducted using individual specimens [7-12] or culture [11] and has focused on comparing the Alinity m STI assay with other commercial tests. To the best of our knowledge, this is the first study to use pooled specimens in the Alinity m STI assay.

As observed in other assays [13], CN values on the Alinity m system tend to be higher than Ct values. However, the Alinity m STI assay demonstrates comparable performance to other molecular tests, such as the GeneXpert CT/NG assay. In a comparative analysis, the mean Ct difference between the two platforms was -1.6 for CT and 0.0 for NG [10].

The pooling of specimens for CT/NG testing can lead to the inhibition of samples because of several factors. Substances present in certain anatomical site samples can interfere with PCR [3,4]. Abbott has reported that CN delays can occur when a sample contains substances such as mucus, seminal fluid, γ-globulin, glucose, Preparation H Hemorrhoidal cream, faeces, and Sensodyne Repair & Protect Sensitive toothpaste [1,2]. These substances may interfere with the assay, particularly at lower target levels, potentially affecting amplification efficiency and delaying detection.

The combination of different sample matrices can increase the likelihood of inhibition, leading to false negatives. However, it has been observed that the dilution effect in the pool can counteract the effect of inhibitors present in the positive samples [3,4]. Our results also confirmed that CT, NG, and MG detection with the Alinity m is possible after pooling. Some tests were inhibited or partially inhibited, indicating the need for new specimens for re-testing. The pooling methodology (ex-ante vs. ex-post pooling) also played a role [3]. In our study, the preliminary validation was successful using post pooling. However, it has been probed that ex-ante pooling (combining samples immediately after collection) is less prone to inhibition compared to ex-post pooling (combining samples after initial individual testing), which, furthermore, can introduce additional handling and storage variables [3]. Pooling can also affect the sensitivity and specificity of CT/NG detection. The bacterial load in the pooled samples varied significantly, affecting the detection rate. Samples with lower bacterial loads were more likely to yield false negatives when pooled [4].

Our study is limited by its retrospective design, and we have yet to determine the detection limit or analytical sensitivity of pooled specimens. Nevertheless, multisite pooled testing and pooled testing from multiple individuals have been used as sensitive and specific methods for CT/NG detection [4,10-16].

The pooling of samples from multiple anatomical sites can affect countries with national guidelines that recommend different dosages and treatment schedules based on the site of infection. This may imply retesting of the samples from each site to prescribe the correct treatment. The collection of new samples from positive individuals can lead to treatment delays and other issues during patient follow-up. These concerns are not applicable in countries following current WHO recommendations for treating genital or anorectal chlamydial infections with doxycycline or gonorrhoea with dual therapy [16]. However, due to increasing resistance to ceftriaxone and azithromycin, WHO is working on updating the treatment guidelines for NG, which may impact treatment practices for infections at various anatomical sites [16].

While testing from individual anatomic sites would be ideal, the increased cost of nucleic acid amplification tests is a limitation, especially in low- and middle-income countries and other resource-limited settings [14]. For example, in Belgium, molecular diagnostic tests for CT, NG, MG, and TV are reimbursed under certain conditions regulated by the national healthcare system. Patients can only receive reimbursement for these tests up to twice per year, depending on specific circumstances such as symptoms or exposure to infection. A study among men who have sex with men reported that triple-site testing (using pharyngeal, rectal, and urethral/first-void urine samples) offers cost savings of up to two-thirds of the costs of the assays alone, as well as savings in consumables, processing time, and clinical pathway efficacy [17]. Similarly, pooling samples from multiple anatomical sites in female sex workers yielded approximately 35% savings in reagent costs and laboratory technician time, with minimal loss in diagnostic sensitivity [18]. Additionally, testing multiple anatomical sites individually can significantly increase costs and workload, particularly when implemented at or near the point of care.

## Conclusions

The Alinity m STI assay was compatible with the pooled samples. In our laboratory, within four years, only 1.8% of the 5324 pooled specimens tested were fully inhibited, indicating that the assay can handle pooled specimens with minimal interference. Using pooled specimens can be helpful for laboratories with budget restrictions and/or where the number of specimens to be tested per patient is restricted, owing to reimbursement or payment policies. Nonetheless, further studies could help refine the understanding of pooled specimen testing in the Alinity m system, especially in terms of detection limits and sensitivity across various anatomical sites.
